# Efficacy of YL-1 hematoma crushing needle combined with hematoma drainage in intracerebral hemorrhage treatment

**DOI:** 10.3389/fmed.2025.1495160

**Published:** 2025-02-26

**Authors:** Xianyong Chen, Danhong Chen, Shaonan Sun, Zhenyong Huang, Weipeng Hu, Qiangbin Zhu

**Affiliations:** ^1^Department of Neurosurgery, Hui’an County Hospital and Hui’an County Hospital Affiliated to Quanzhou Medical College, Quanzhou, Fujian, China; ^2^Department of Intensive Care Unit, Hui’an County Hospital and Hui’an County Hospital Affiliated to Quanzhou Medical College, Quanzhou, Fujian, China; ^3^Department of Neurosurgery, The Second Affiliated Hospital of Fujian Medical University, Quanzhou, China

**Keywords:** intracerebral hemorrhage, YL-1 hematoma crushing needle, hematoma drainage, craniotomy, urokinase, minimally invasive surgery

## Abstract

**Objective:**

Early craniotomy evacuation in hematoma surgery does not significantly improve the prognosis of patients with spontaneous intracerebral hemorrhage (ICH). The YL-1 hematoma crushing puncture needle, specifically designed for ICH evacuation, has an uncertain therapeutic efficacy. This study aimed to evaluate its clinical effectiveness.

**Materials and methods:**

We retrospectively reviewed medical records of patients with ICH who underwent twist intraosseous drill needle (TIDN) surgery at our institution between September 2016 and March 2023. Clinical outcomes were analyzed.

**Results:**

The surgical group demonstrated a significantly shorter hematoma resolution time, averaging 14.71 days less than the conservative group (*p* < 0.001). The Barthel Index improved more in the surgical group, with an average increase of 8.214 points (*p* < 0.001). Seven days post-admission, the increase in Glasgow Coma Scale (GCS) scores was significantly higher in the surgical group, with an average improvement of 1.471 points (*p* < 0.001). Additionally, the duration of mannitol administration was significantly reduced in the surgical group (*p* < 0.001).

**Conclusion:**

TIDN surgery combined with hematoma drainage may serve as a viable surgical alternative for basal ganglia hemorrhage patients. This approach appears to reduce mannitol usage, mitigate craniotomy-associated risks, and promote short-term improvements in GCS scores and Barthel Index, highlighting its potential clinical benefits.

## Introduction

Spontaneous intracerebral hemorrhage (ICH) is a common neurological condition associated with high mortality (1). Surgical interventions for ICH include craniotomy for hematoma evacuation, neuroendoscope-assisted hematoma removal, and stereotactic minimally invasive hematoma puncture with drainage (2). However, early craniotomy evacuation has shown limited improvement in patient prognosis (2, 3). With the increasing adoption of minimally invasive techniques, there is a growing preference for such approaches in ICH management (4–6). The YL-1 hematoma crushing needle, also known as the twist intraosseous drill needle (TIDN), is a specialized device designed for intracranial hematoma evacuation (7, 8). TIDN surgery is minimally invasive, technically simple, and may offer a viable option for patients unable to tolerate craniotomy (9). While it has been used in the treatment of chronic subdural hematomas (10–12), its efficacy in ICH treatment remains uncertain. To evaluate the effectiveness of TIDN surgery using the YL-1 hematoma crushing needle in ICH management, we conducted a retrospective analysis of 51 patients with spontaneous ICH admitted to our institution between September 2016 and March 2023.

## Materials and methods

The diagnosis of ICH was confirmed through head CT imaging. Additionally, Computed Tomography Angiography (CTA) or Digital Subtraction Angiography (DSA) scans were performed to exclude vascular abnormalities such as intracranial aneurysms and arteriovenous malformations. All patients were diagnosed and treated in strict accordance with stroke treatment guidelines and underwent minimally invasive puncture combined with hematoma drainage. TIDN surgery was performed only after obtaining written informed consent from the patient’ s legal guardian or next of kin, including specific consent for urokinase administration. This study was approved by the institutional ethics committee (approval number: 2025001), and informed consent was obtained from all participants in accordance with ethical guidelines.

### Inclusion criteria

(1) Age ≥ 40 years. (2) Diagnosis of hypertensive intracerebral hemorrhage (ICH) confirmed by head CT scan, with symptom onset within 24 h. (3) The hematoma must be located in the basal ganglia, with or without intraventricular hemorrhage. (4) Hematoma volume > 25 mL without signs of brain herniation. (5) CTA or DSA performed to exclude vascular abnormalities such as intracranial aneurysms and arteriovenous malformations.

### Exclusion criteria

(1) Intracerebral hemorrhage caused by intracranial aneurysms, arteriovenous malformations, tumors, infarction, or trauma. (2) Severe coagulopathies (e.g., thrombocytopenia, hepatitis) or severe dysfunction of the heart, liver, kidneys, or lungs. (3) History of stroke leading to neurological deficits, discontinuation of treatment, or death after admission. (4) Refusal to sign the informed consent form.

### Clinical data

Baseline clinical data included age, sex, Glasgow Coma Scale (GCS) scores at admission and on the seventh day of hospitalization, Barthel Index scores at admission and discharge, hematoma volume at admission, hematoma resolution time, mannitol usage, and length of hospital stay. Additionally, potential prognostic factors were considered, including comorbidities, complications, personal medical history, past medical history, and anticoagulant use. TIDN surgery was performed only after obtaining written informed consent from the patient’s legal agent or authorized representative, specifically including consent for urokinase administration. The procedure utilizing the YL-1 hematoma crushing needle kit has been comprehensively detailed in our previous report (8). The general surgical steps were as follows.

### Surgical steps

**FIGURE 1:**
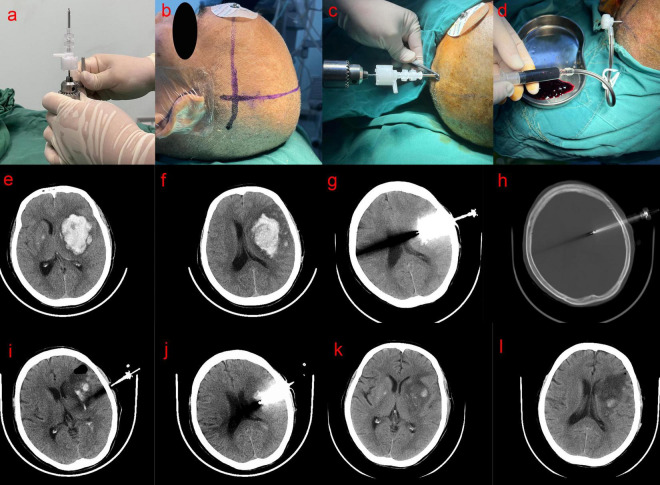
**(a)** Mounting of the YL-1 hematoma crushing needle on the drill; **(b)** positioning puncture point; **(c)** perpendicular drilling **(d)** aspirated; **(e,f)** preoperative head CT scan showed the hematoma volume about 45 mL; **(g,h)** CT scan on the first day after operation showed that the YL-1 puncture needle penetrated the skull into the hematoma cavity; **(i,j)** CT scan on the 4th day after operation showed that hematoma was almost evacuated; **(k,l)** 10 days after the operation, head CT showed that the hematoma was completely evacuated.

**Fixation of the YL-1 Hematoma Crushing Needle:** The YL-1 hematoma crushing needle was securely attached to a handheld electric drill (Figure 1a).

**Localization:** Anatomical landmarks near the hematoma site and CT-based markers were used to determine the hematoma plane (Figure 1b). The scalp overlying the largest layer of the hematoma, as identified on CT scans, was selected as the puncture site, ensuring the avoidance of the Sylvian fissure. The intracranial and extracranial distance from the hematoma center to the puncture point was measured using CT imaging, and a 5.0 cm or 7.0 cm YL-1 hematoma crushing needle kit (Beijing Wantefu Medical Equipment Co., Ltd.) was selected accordingly.

**Puncture and Aspiration:** The patient was placed in a supine position, and the surgical field was disinfected with 2% iodine tincture, followed by deiodination with 75% alcohol after 2–3 min, before applying a sterile drape. After administering local anesthesia with 5 mL of 2% lidocaine to the scalp and periosteum, an assistant stabilized the patient’s head. The needle was inserted perpendicularly to the cranial surface at the preselected puncture site without making a skin incision (Figure 1c). A distinct breakthrough sensation was felt upon penetrating the inner table of the skull. At this point, the YL-1 puncture needle was detached from the drill and automatically fixed onto the skull. Next, the depth of the YL-1 puncture needle was adjusted. The drill cap was removed, and a plastic sleeve needle was manually inserted, advancing the entire assembly slowly until reaching the predetermined depth. This ensured that the front end of the hematoma crushing needle was positioned at the center of the hematoma cavity. The plastic inner needle was then removed, and the three-way needle body was sealed with a cap and gasket. A drainage tube was connected, and hematoma aspiration was performed using a 5-mL syringe (Figure 1d). After aspirating the liquid portion of the hematoma, a drainage device was connected to the tubing. Since hematomas often contain solid components, complete aspiration was not always achieved during the initial attempt.

**Postoperative Management and Urokinase Administration:** Drainage volume was recorded, and a follow-up head CT scan was performed within 24 h postoperatively to assess the need for urokinase injection for hematoma liquefaction and drainage. The urokinase dosage was determined based on the method described by Liu et al. (13): 20,000 IU of urokinase (Tianjin Biochemical Pharmaceutical Co., Ltd.) diluted in 5 mL of normal saline. Following urokinase injection, the drainage tube was clamped for 2–4 h before reopening. Depending on the drainage volume, urokinase was administered continuously for up to 3 days. On postoperative day 3, a follow-up head CT scan was conducted. If significant hematoma remained, bedside aspiration or continued urokinase perfusion was considered. To minimize the risk of intracranial infection, urokinase administration did not exceed 5 days.

Figures 1e–l illustrate preoperative and postoperative CT scans of a representative case.

### Primary endpoint

**Hematoma Volume Calculation:**

Hematoma volumes were calculated using the Tada formula (14):

Hematoma volume = $\frac{\pi }{6}$ × length diameter (cm) × width diameter (cm) × layer thickness (cm) × number of layers.

**Hematoma Disappearance Assessment:**

Hematoma disappearance was defined as follows: if the ratio of residual hematoma volume to initial hematoma volume, multiplied by 100%, was <5%, it was considered that the hematoma had disappeared.

**Activities of Daily Living (ADL) Assessment:**

The patient’s activities of daily living (ADL) were assessed using the Barthel index for ADL scale (15). ADL improvement amplitude (Δ ADL) was calculated as:

Δ ADL = Barthel index at discharge - Barthel index at admission

A negative value indicated a decrease in ADL.

**Increase in Glasgow Coma Scale (GCS) Score After 7 Days:**

The increase in GCS score after 7 days of admission (Δ GCS) was calculated as:

Δ GCS = GCS score on the 7th day after admission – GCS score at admission

A negative value indicated a decrease in the GCS score.

**Mannitol Administration:**

Mannitol was administered in accordance with the manufacturer’s instructions. The dose of mannitol was calculated based on the standard of 1.5 g⋅kg-1⋅d-1, which is equivalent to 125 mL of 20% mannitol intravenous drip per day for adults weighing 50 kg, administered once every 8 h. Mannitol duration (in days) was calculated as:

Mannitol Duration (days) = (Total Mannitol Use)/(1.5 g⋅kg-1⋅d-1))

### Statistical analysis

Statistical analysis was performed using IBM SPSS software (version 23.0, IBM; Armonk, NY, USA). Descriptive statistics were expressed as mean ± standard deviation ($\bar{\text{x}}$ ± s) for continuous variables and percentage (%) for categorical variables.

The normality of continuous variables was assessed using the Shapiro-Wilk test. If the data followed a normal distribution, independent *t*-tests were used to compare means between two groups. For non-normally distributed data, the Mann-Whitney *U* test was applied for comparisons between two independent samples. Categorical variables were analyzed using the chi-square test. Statistical significance was set at *p* < 0.05.

## Results

From September 2016 to April 2023, a total of 420 patients with intracerebral hemorrhage (ICH) were admitted to our institution, including 352 patients with hematomas located in the basal ganglia. Of these, 153 patients underwent surgical treatment: 47 received craniotomy, 45 underwent surgery using the YL-1 hematoma crushing needle, and 61 had nerve endoscope-assisted hematoma evacuation or other minimally invasive hematoma drainage surgeries. A total of 51 patients met the inclusion criteria, with 30 receiving surgical treatment using the YL-1 hematoma crushing needle and 21 undergoing conservative treatment.

In the surgical group, there was one case each of mild pulmonary dysfunction (bronchiectasis), hypertensive heart disease, and senile heart valve disease. In the conservative treatment group, four patients had mild pulmonary dysfunction (all due to senile emphysema), one patient had hypertensive heart disease, and one had atrial fibrillation.

Imaging Characteristics: All patients underwent head CT scans upon admission. Hematomas were located in the basal ganglia in all cases. In the surgical group, four cases had intraventricular hemorrhage, while two cases in the conservative treatment group had intraventricular hemorrhage.

Medical History: In the surgical group, two patients had a history of anticoagulant use, while no patients in the conservative group had such a history. Patient information is summarized in Table 1.

**TABLE 1:** Comparison of data between the surgical group and the conservative group.

	Surgical group (*n* = 30)	Conservative group (*n* = 21)	Mean value difference	Difference 95 % confidence interval		*F*	t/χ ^2^	*P*
				**Lower**	**Upper**			
Age (y)	60.967 ± 9.960	61.095 ± 10.227	−0.129	−5.886	5.629	0.000	−0.045	0.964
Males	17 (56.7%)	13 (61.9%)					0.140	0.778
Females	13 (43.3%)	8 (38.1%)					0.140	0.778
Comorbidities	3 (10.0%)	5 (23.8%)					1.781	0.182
Smoking history	3 (10.0%)	1 (4.8%)					0.469	0.493
Initial GCS	10.133 ± 2.70	11.667 ± 2.887	−1.533	−3.122	0.055	0.194	−1.940	0.058
Initial ICH volume (mL)	44.443 ± 14.329	38.000 ± 9.813	6.443	−0.808	13.694	2.879	1.786	0.080
ΔGCS	1.567 ± 1.716	0.095 ± 1.179	1.471	0.60247	2.340	4.301	3.403	0.001
Time of hematoma disappearance (d)	9.100 ± 3.871	23.810 ± 3.803	−14.710	−16.907	−12.512	0.070	−13.450	<0.001
ΔADL	25.833 ± 10.755	17.619 ± 13.098	8.214	1.486	14.943	2.822	2.453	0.018
Hospitalization days (d)	33.467 ± 12.760	33.000 ± 12.857	0.467	−6.852	7.785	0.165	0.128	0.899
Complications	22 (73.3%)	11 (52.4%)					2.375	0.123

GCS, Glasgow Coma Scale score; ICH, intracerebral hemorrhage; ADL, Activities of Daily Living.

Regarding the average age, the surgical group had an average age of 60.97 ± 9.96 years, while the conservative group had an average age of 61.10 ± 10.23 years. The independent sample *t*-test yielded *t* = −0.045, *p* > 0.05, indicating no statistically significant difference.

When comparing the sex distribution between the surgical and conservative groups, the surgical group consisted of 17 males (56.7%) and 13 females (43.3%), while the conservative group included 13 males (61.9%) and 8 females (38.1%). The chi-square test results revealed χ^2^ = 0.140, *p* > 0.05, indicating no statistically significant difference.

When comparing the distribution of comorbidities between the surgical and conservative groups, 3 patients (10.0%) in the surgical group had comorbidities, while 5 patients (23.8%) in the conservative group had comorbidities. The chi-square test results showed χ^2^ = 1.781, *p* > 0.05, indicating that the difference was not statistically significant.

When comparing the distribution of smoking history between the surgical and conservative groups, 3 patients (10.0%) in the surgical group had a smoking history, while 1 patient (4.8%) in the conservative group had a smoking history. The chi-square test results showed χ^2^ = 0.469, *p* > 0.05, indicating that the difference was not statistically significant.

For the average hematoma volume at admission, the surgical group had an average volume of 44.44 ± 14.33 mL, while the conservative group had an average volume of 38.00 ± 9.81 mL. The independent sample *t*-test showed *t* = 1.786, *p* > 0.05, indicating no statistically significant difference.

In terms of Glasgow Coma Scale (GCS) scores at admission, the surgical group had an average GCS score of 10.13 ± 2.70, while the conservative group had an average GCS score of 11.67 ± 2.89. The independent sample *t*-test revealed *t* = −1.940, *p* > 0.05, suggesting no statistically significant difference.

When comparing the average ΔADL between the surgical and conservative groups, the results indicated that the surgical group had a significantly higher ΔADL (25.83 ± 10.76) compared to the conservative group (17.62 ± 13.10), with an average increase of 8.21 (95% CI 1.49 – 14.94). The independent sample *t*-test results revealed *t* = 2.453, *p* = 0.018 < 0.05, indicating a statistically significant difference.

Regarding the average ΔGCS, the surgical group showed a significantly higher ΔGCS (1.57 ± 1.72) compared to the conservative group (0.10 ± 1.18), with an average increase of 1.47 (95% CI 0.60–2.34). The independent sample *t*-test results showed *t* = 3.403, *p* = 0.001 < 0.05, indicating a statistically significant difference.

For the average hospitalization duration, the surgical group had a mean length of stay of 33.47 ± 12.76 days, while the conservative group had a mean of 33.00 ± 12.86 days. The independent sample *t*-test results showed *t* = 0.128, *p* > 0.05, indicating no statistically significant difference between the two groups.

When comparing the average time for hematoma disappearance, the surgical group (9.10 ± 3.87 days) had a significantly shorter duration than the conservative group (23.81 ± 3.80 days), with an average reduction of 14.71 days (95% CI 12.51–16.91). The independent sample *t*-test results showed *t* = −13.45, *p* < 0.001, indicating a statistically significant difference.

The Mann–Whitney U test was used to assess the difference in the duration of mannitol administration between the surgical and conservative groups. The average rank for mannitol duration in the surgical group was 19.170, while the conservative group had an average rank of 35.760. The Mann-Whitney U test results indicated a statistically significant difference in the duration of mannitol between the two groups (*U* = 110.00, *p* < 0.001).

When comparing the distribution of complications between the surgical and conservative groups, 22 patients (73.3%) in the surgical group experienced complications, including 21 cases of lung infection and 1 case of stress ulcer. In the conservative group, 11 patients (52.4%) had complications, all of which were lung infections. The chi-square test results showed χ^2^ = 2.375, *p* > 0.05, indicating that the difference was not statistically significant.

## Discussion

This study compared the baseline characteristics between the surgical and conservative groups, including age, sex, comorbidities, medical history, GCS score at admission, and initial hematoma volume. No significant differences were observed between the groups (*p* > 0.05).

The surgical group demonstrated a significantly shorter hematoma resolution time compared to the conservative group, with an average reduction of 14.71 days (*p* < 0.001). Additionally, the surgical group showed a greater increase in the Barthel index (ΔADL), with an average improvement of 8.21 points (*p* < 0.001). After 7 days of admission, the increase in GCS scores was also significantly higher in the surgical group, with an average improvement of 1.47 points (*p* < 0.001). Furthermore, the amount of mannitol administered in the surgical group was significantly lower than in the conservative group (*p* < 0.001).

However, there were no significant differences in the length of hospital stay between the two groups (*p* > 0.05), and surgery did not significantly increase the incidence of complications (*p* > 0.05).

TIDN surgery is a simple and cost-effective procedure that requires only local anesthesia at the puncture site, resulting in a short surgical duration and minimal bleeding. The procedure utilizes a YL-1 hematoma crushing needle and does not require a scalp incision. Instead, a small bone hole, approximately 2 mm in diameter, is drilled into the skull, allowing for self-fixation (8). This approach is particularly advantageous for older patients or those with varying severities of underlying conditions who may not tolerate a craniotomy. The use of the YL-1 hematoma crushing needle for puncture and drainage reduces surgical trauma (9, 16). Additionally, older individuals often experience varying degrees of brain atrophy, which leads to relatively larger cranial spaces (17). Compared to younger patients, older individuals have a larger intracranial buffer space when their brain tissue is compressed by the same hematoma volume, providing favorable conditions for minimally invasive puncture and drainage surgery.

Urokinase is a promising fibrinolytic agent known for its safety and efficacy (18). It not only enhances the clot dissolution rate but also reduces the adverse outcomes associated with fibrinolysis following ICH (19, 20). Recent studies report that the risk of rebleeding associated with urokinase ranges from 4.4 to 19.4% (18, 21, 22). Consistent with previous findings (8), the urokinase dose administered in this study was 20,000 UI/d. Following 3 days of urokinase treatment, a follow-up head CT scan was conducted. Based on the residual hematoma volume, a decision was made whether to proceed with secondary aspiration or to re-administer urokinase for further hematoma liquefaction. Most intracranial hematomas were evacuated after 5 days of minimally invasive puncture and drainage; thus, urokinase is typically used for no more than 5 days (13). In our experience, after 3 days of urokinase treatment, the hematoma becomes gelatinous and can be easily aspirated. If the head CT scan shows a significant residual hematoma, a 5-mL syringe can be used to aspirate the hematoma twice through the drainage tube. After the second aspiration, most hematomas can be successfully evacuated. It is crucial to note that excessive aspiration may lead to rebleeding.

Following intracerebral hemorrhage (ICH), in addition to the destruction of neurons at the lesion site, secondary neuronal injury around the lesion begins in the early stages of the disease. Inflammation, thrombin activation, and erythrocyte dissolution resulting from hematoma formation can promote the development of cerebral edema (23, 24). Early hematoma evacuation is crucial to alleviate the compression on adjacent brain tissue, potentially reducing secondary neuronal injury (25, 26). In this study, the hematoma resolution time in the surgical group was shorter than in the conservative group. Early evacuation of some hematomas in the surgical group facilitated partial decompression, allowing patients to pass the peak of the intracranial pressure-volume curve earlier and preventing progression to cerebral herniation. After 7 days of treatment, the increase in GCS scores in the surgical group was 1.471 points higher than in the conservative group. This improvement may be attributed to the reduction of secondary neuroinflammation following hematoma evacuation, thereby shortening the duration of cerebral edema and enhancing neurological recovery in ICH patients.

Perihematomal edema (PHE) can develop following intracerebral hemorrhage (ICH). PHE gradually increases within the first 24 h and intensifies rapidly 3 days after disease onset. It typically reaches its initial peak between the 7th and 11th day, followed by a slow, sustained increase (27). Twenty percent mannitol is the preferred hypertonic agent for clinical management of elevated intracranial pressure (28, 29). In patients without contraindications to mannitol, this treatment alone may suffice to surpass the peak of the intracranial pressure-volume curve. However, some patients may have underlying cardiac or renal dysfunction, and a rapid increase in blood volume following mannitol administration could lead to congestive heart failure. Consequently, excessive fluid intake must be avoided, and patients with severe renal insufficiency should restrict fluid intake to prevent exacerbation of PHE (30). In such cases, the limitation of dehydrating agents may impair PHE resolution and worsen the prognosis. In this study, the duration of mannitol use was reduced in the surgical group, with some patients not requiring mannitol for PHE treatment post-surgery. Given that the majority of ICH patients are older adults with varying degrees of cardiopulmonary insufficiency and other comorbidities, the risks associated with craniotomy and the size of the surgical wound are increased (31). The YL-1 hematoma crushing needle, which requires only local anesthesia, enables hematoma puncture and drainage with a smaller surgical wound, less bleeding, and a shorter operation time (8, 9). Therefore, the use of the YL-1 hematoma crushing needle for hematoma puncture and drainage represents a surgical method worth considering.

TIDN surgery is convenient, quick, and more cost-effective than traditional craniotomy. Conventional hematoma drainage surgery involves making an incision in the scalp and drilling a hole in the skull to create a puncture channel (32, 33). In contrast, TIDN surgery eliminates the need for a scalp incision, allowing for direct drilling through both the skull and dura mater, which facilitates rapid puncture to the target site. This approach minimizes trauma, reduces blood loss, and streamlines the surgical procedure. It enables effective removal of intracranial hematomas while avoiding craniotomy, which can reduce the financial burden on patients. For those undergoing anticoagulant therapy, the risk associated with craniotomy is significantly increased. Our preliminary experience supports the use of TIDN in patients on anticoagulants, as it offers potential benefits for these individuals (9). In this study, two patients receiving anticoagulant therapy showed positive treatment outcomes following TIDN surgery. Although the sample size is small, these preliminary results suggest that TIDN surgery may have potential applications in patients undergoing anticoagulant therapy. However, these findings require validation through larger-scale studies to confirm their efficacy and safety in clinical practice.

### Limitations

Our study has several limitations. First, we did not compare TIDN with other surgical approaches such as craniotomy hematoma evacuation, nerve endoscope-assisted hematoma evacuation, or stereotactic hematoma puncture drainage. Therefore, a large-scale, multicenter, randomized controlled trial (RCT) is necessary to further evaluate these methods. Second, this study focused exclusively on patients with basal ganglia hemorrhage. Future research should explore the efficacy of TIDN in treating intracerebral hemorrhage (ICH) in other brain regions to better understand its broader applicability and potential benefits. Third, as a single-center retrospective study, our findings are subject to inherent biases and limitations associated with retrospective analyses and single-center samples. Multi-center prospective studies or RCTs would provide more robust evidence, and future research should consider adopting these designs to validate our results. Finally, the lack of long-term follow-up data and information on surgical complications limits the comprehensiveness of our analysis. Future studies should incorporate these data to fully assess the long-term efficacy and safety of the YL-1 hematoma crushing needle.

## Conclusion

The combination of TIDN puncture with hematoma drainage offers a safe, effective, and minimally invasive surgical alternative for older patients with basal ganglia hemorrhage who are unsuitable for craniotomy. The use of a YL-1 hematoma crushing needle in TIDN treatment appears to reduce the need for mannitol administration while avoiding craniotomy. Additionally, this approach may lead to short-term improvements in patients’ GCS scores and Barthel index.

## Data Availability

The raw data supporting the conclusions of this article will be made available by the authors, without undue reservation.
